# One-Step Synthesis of Cytocompatible Brushite-Mineralized Gellan Gum/Alginate Microgels via a Temperature-Controlled Approach

**DOI:** 10.1021/acsomega.5c12166

**Published:** 2026-06-22

**Authors:** Stanisław Marecik, Anna Kusibab, Krzysztof Pietryga, Elżbieta Pamuła

**Affiliations:** † 49811 AGH University of Krakow, Faculty of Materials Science and Ceramics, Department of Biomaterials and Composites, al. A. Mickiewicza 30, Kraków 30-059, Poland; ‡ Silesian Park of Medical Technology Kardio-Med Silesia, Marii Curie-Skłodowskiej 10C, Zabrze 41-800, Poland

## Abstract

Injectable bioactive microgels (MGs) are in high demand in minimally invasive bone regeneration, but their fabrication presents significant challenges. Specifically, creating composite MGs that are structurally stable—requiring high polymer concentrations—and containing a bioactive mineral phase is technologically difficult due to high viscosity, leading to inconsistent particle formation and system clogging. Our objective was to develop a novel temperature-controlled emulsification method to overcome these limitations and to produce and characterize MG from a brushite-mineralized highly concentrated gellan gum/sodium alginate (GG/SA). The setup allowed us to successfully produce uniform spherical MG with a controllable mineral content of up to 30% and a mean diameter below 100 μm. SEM/EDS, FTIR, XRD, and TG analyses confirmed the successful incorporation of a nanocrystalline brushite phase, which provided significant structural stability to MGs. *In vitro* assays demonstrated that all MGs are cytocompatible with MG63 osteoblast-like cells. Cell culture experiments in dynamic conditions revealed that the mineralized MGs support cell adhesion and spreading, contrasting with the nonmineralized controls, where no cell anchorage was observed. These findings demonstrate that precise temperature control is a successful strategy for processing high-viscosity GG/SA solutions into uniform MGs. The resulting materials combine the structural and bioactive benefits of a nanocrystalline brushite phase with the established biocompatibility of a GG/SA matrix. Ultimately, this work establishes an accessible and versatile temperature-controlled fabrication platform. While successfully demonstrated here for GG/SA/brushite composites, this setup can be broadly applied to process other high-viscosity, thermoresponsive biopolymers, opening new avenues for the tunable production of advanced MGs in tissue engineering.

## Graphical abstract

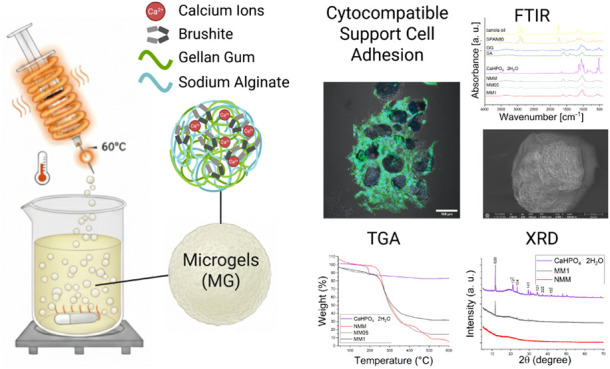

## 1 Introduction

Bone tissue defects resulting from trauma, osteoporosis, and tumor resection pose significant medical challenges.^1^ This is because any bone defect with a diameter greater than the critical size, which in some cases is only 2 cm in humans, requires medical intervention to achieve successful regeneration.^2–4^ Consequently, there is a significant demand for biomaterials that can serve as fillers for such defects. These biomaterials should ideally not only provide structural support^1^ but also actively promote bone tissue regeneration^5,6^ and be applicable in a minimally invasive manner, such as through injection.^7–9^ Furthermore, these materials must be biocompatible and preferably biodegradable^5^ to ensure that they can be safely integrated into the body and slowly degrade without causing harmful effects.

In recent years, hydrogel-based systems, particularly in the form of microgels, have gained considerable attention for bone tissue engineering.^10–12^ Their remarkable ability to absorb and retain large amounts of aqueous media makes them effective carriers for biologically active substances, such as drugs or growth factors.^13,14^ Furthermore, their micrometer-scale size and spherical shape result in a high surface-to-volume ratio.^10^ These characteristics facilitate the efficient exchange of nutrients, oxygen, and therapeutic agents while providing a larger surface area for cell interaction. Furthermore, microgel suspensions can exhibit favorable rheological properties, such as shear thinning, allowing for injectability^15,16^ and precise adaptation to the irregular shapes of implantation sites, further enhancing their potential for biomedical applications.^17,18^

Microgels can be synthesized from a variety of natural and synthetic polymers. Among natural polymers, polysaccharides are frequently chosen because of their inherent biocompatibility, biodegradability, and structural similarity to the extracellular matrix. This study uses a composite system of gellan gum (GG), produced by the bacterium *Sphingomonas elodea*, and sodium alginate (SA), extracted from brown algae, which are two anionic polysaccharides. While widely recognized as noncytotoxic and biotolerable,^19–22^ these polymers are inherently bioinert and lack specific cell-adhesion motifs, which often limits initial cell anchorage without further modification.^23^ While both polymers undergo ionotropic gelation in the presence of divalent cations such as Ca^2+^ to form stable networks, their specific mechanisms differ. Sodium alginate is valued for its gentle, temperature-independent cross-linking with Ca^2+^.^24^ In contrast, the formation of mechanically robust gellan gum hydrogels requires a synergistic two-step process: a temperature-induced coil-to-helix transition upon cooling, followed by the essential ionic cross-linking of these helical structures by cations. While sodium alginate is frequently used for microgel fabrication at room temperature, it often yields hydrogels with insufficient structural stability and stiffness for bone regeneration applications. To overcome this obstacle, we incorporated GG, which is known to form mechanically robust networks. However, processing highly concentrated GG solutions requires elevated temperatures to maintain a low viscosity, a condition that poses a significant technological challenge for conventional microgel fabrication systems. Although the combination of these polymers offers superior physicochemical properties, processing such a viscous blend poses a significant technological challenge for conventional emulsification systems.

GG and SA hydrogels serve as an excellent organic framework for mineralization, a key strategy to enhance the osteogenic potential of biomaterials.^25–30^ This approach aims not only to mimic the final composition of bone but also to mimic the dynamic processes of natural biomineralization. For this reason, this study focuses on the mineralization of GG/SA microgels using calcium hydrogen phosphate dihydrate (CaHPO_4_·2H_2_O), commonly known as brushite.

Brushite emerges as a promising bioactive precursor material.^31^ Its advantage over hydroxyapatite, a material widely used for bone regeneration as the gold standard, stems from its much higher solubility. This is reflected in its pKsp, i.e., the constant negative logarithm of the solubility product, of approximately 6.6 at 25 °C, compared to the pKsp of hydroxyapatite, which is 116.6 at 25 °C.^32^ From the perspective of bone regeneration, the much higher solubility of brushite under physiological conditions is of key advantage. This makes it an inherently more bioresorbable material that acts as a readily available source of calcium and phosphate ions, essential building blocks for new bone tissue. In contrast, hydroxyapatite, being a thermodynamically stable and far less soluble phase, behaves more like a permanent implant than a dynamic participant in the healing process.^33^ Furthermore, this characteristic enables the biomimetic transformation of brushite into nanocrystalline apatite, a process that mimics natural mineralization pathways.^34,35^ The local supersaturation of ions in the cellular microenvironment triggers specific osteoinductive mechanisms that go beyond passive osteoconduction. The released Ca^2+^ act as signaling molecules, activating the calcium-sensing receptor (CaSR) on the surface of progenitor cells.^36^ This initiates intracellular cascades, such as the mitogen-activated protein kinase (MAPK) pathway, leading to increased expression of key osteogenic transcription factors such as runt-related transcription factor 2 (Runx2) and Osterix (Osx).^37,38^ Simultaneously, phosphate ions transported into cells stimulate key metabolic processes, including the synthesis of adenosine triphosphate (ATP).^39^ This, in turn, acts as a signal that directs stem cells to the osteogenic lineage and is essential for matrix mineralization.^39^ Furthermore, the same ions exhibit osteoimmunomodulatory capabilities, promoting macrophage polarization toward a proregenerative M2 phenotype.^40^ Its macrophage-driven resorption occurs at a rate that can be synchronized with the healing phases, efficiently creating space for the formation of new tissue.^41^ These characteristics position brushite as an active, rather than inert, participant in the bone regeneration process.

Despite the clear advantages of the individual components, combining them into a single injectable biomaterial poses significant technological challenges. Fabricating robust, mineralized microgels from high-concentration polysaccharide solutions is notoriously difficult. Although room-temperature emulsification is suitable for simple alginate systems, it is inadequate for high-concentration gellan gum, which requires elevated temperatures to prevent premature gelation and system clogging.^42^ Therefore, there is a critical need for a fabrication method that can handle high-viscosity, temperature-sensitive polymer solutions while simultaneously incorporating a bioactive mineral phase in a single step. To address these limitations, we developed a novel water-in-oil (w/o) emulsification method centered on a custom-designed temperature control system for the syringe and needle, which enables the processing of high-concentration gellan gum solutions to produce microgels that are smaller and more uniform than previously reported.^42–44^ The aqueous phase, which contains GG, SA, and mineral precursors, was emulsified in a continuous phase of canola oil with sorbitan monooleate as a surfactant. Therefore, the primary objective of this work was to establish an accessible, temperature-controlled fabrication platform capable of processing high-viscosity, thermoresponsive polymers. To validate the versatility of this setup, we aimed to (1) optimize the synthesis protocol for a challenging, highly concentrated gellan gum/alginate blend, (2) perform a comprehensive physicochemical characterization of the resulting brushite-mineralized microgels, and (3) conduct an *in vitro* biological evaluation of their cytocompatibility.

## 2 Materials and Methods

### 2.1 Materials

Gellan gum powder (GG, Gelzan, G1910), alginic acid sodium salt powder (SA, 180947), and sorbitan monooleate (SPAN80) were purchased from Sigma-Aldrich, Germany. Calcium chloride (CaCl_2_·2H_2_O) and disodium phosphate (Na_2_HPO_4_), were purchased from Avantor Performance Materials, Poland. Canola oil was purchased from Bunge Polska sp. z o. o., Poland.

To obtain microgels (MGs) with w/o emulsification, a custom-constructed device was used (Figure 1A). This device consisted of a syringe pump, built based on an open-source project,^45^ and a custom-built temperature control system (Figure 1B). The control system was developed to precisely maintain the temperature of the syringe, needle, and beaker. It was based on an ESP32 microcontroller that runs custom software written in C++.^46^ The temperature was monitored by digital sensors and regulated by 40 W/24 V heaters, achieving a thermal stability of ±0.1 °C.

**Figure 1:**
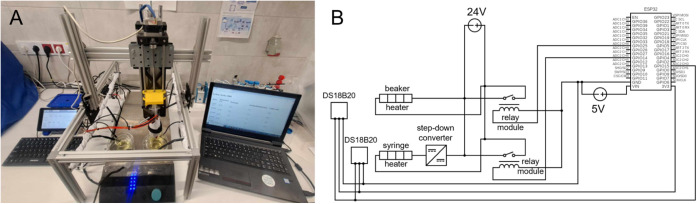
Custom-constructed device (A) and electronic scheme of the temperature control system (B) for microgel (MG) manufacturing.

### 2.2 Preparation of Mineralized GG/SA Microgels

All procedures for MG synthesis were performed under aseptic conditions within a laminar flow cabinet, and all glassware, magnetic stirrers, and other equipment in contact with the materials were sterilized by autoclaving prior to use. The aqueous phase was prepared by dissolving Na_2_HPO_4_ in water to obtain a 50 mM or 100 mM solution, which was then sterilized by filtration through a 0.22 μm syringe filter. This solution was then used to prepare separate solutions of GG and SA. Specifically, 50 mg of GG was dissolved in 1.5 mL of Na_2_HPO_4_ solution, and 10 mg of SA was dissolved in 0.5 mL of Na_2_HPO_4_ solution. The GG solution was heated to 90 °C for 30 min and then cooled to 70 °C before the SA solution was added. Two mineralized samples, designated MM50 and MM100, were prepared using an aqueous phase containing 2.5% (w/v) GG and 0.5% (w/v) SA. The samples were differentiated by the molar concentration Na_2_HPO_4_, which was 50 mM for MM50 and 100 mM for MM100. The oil phase consisted of a 1% (v/v) SPAN80 solution in canola oil.

The aqueous phase (2 mL, 60 °C) was introduced into the oil phase (50 mL, 60 °C) under continuous mixing at 1000 rpm using a magnetic stirrer, with a flow rate of 2 mL/min, using a syringe pump. The resulting emulsion was then stirred at 60 °C for 60 min, subsequently passively cooled to 35 °C, and supplemented dropwise with 2 mL of a 1 M CaCl_2_ solution. The stirring was continued for an additional 10 min at 35 °C. The stirring was then stopped, and the emulsion was stored at 4 °C overnight to allow for complete gelation of the MG. After repeated washing with a 30% isopropanol solution and Milli-Q water, the samples intended for physicochemical characterization were freeze-dried (Alpha 1–2 LDplus, Martin Christ, Germany). The process of MG manufacturing is presented in Figure 2.

**Figure 2:**
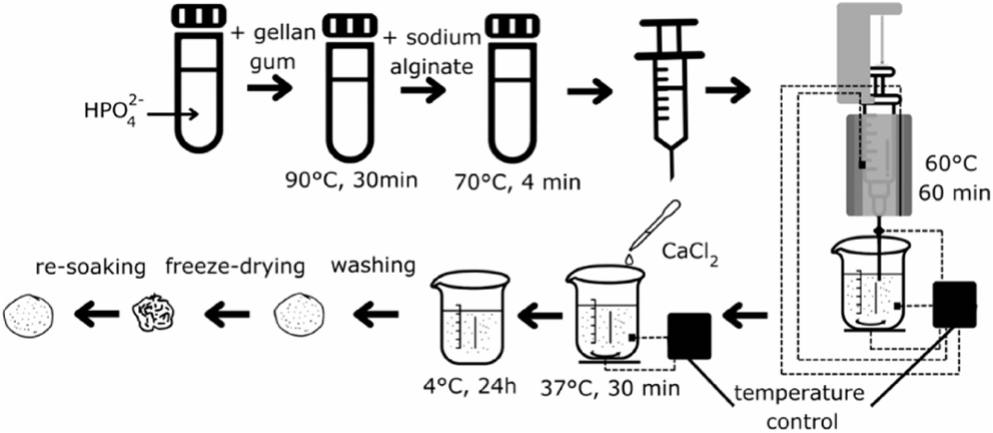
Schematic of the one-step fabrication of brushite-mineralized gellan gum (GG)/sodium alginate (SA) microgels (MGs). The process involves temperature-controlled (60 °C) emulsification of the polymer solution, followed by ionotropic cross-linking with CaCl_2_, washing, and freeze-drying of the final product.

Nonmineralized MG (NMM) was obtained in a similar way, but the polymer solution was prepared with Milli-Q water instead of of Na_2_HPO_4_ solution.

### 2.3 Optical Microscopy and Size Distribution

The morphology of the MG was examined by using an optical microscope (VHX-900F, Keyence, Belgium). MGs were imaged with a digital camera and observed under an optical microscope to assess their shape and size. Their diameter was measured (*n* = 500) using ImageJ software.

### 2.4 Scanning Electron Microscopy and Energy-Dispersive X-ray Spectroscopy

To preserve the spherical morphology of MGs in its hydrated state, the samples were first chemically fixed prior to dehydration and drying. MGs were fixed in 2.5% glutaraldehyde in PBS (pH 7.2) for 2 h at room temperature. Subsequently, the samples were rinsed with PBS and dehydrated through a graded series of ethanol (10%, 25%, 70%, 90%, and 100%), each step lasting 5 min. Finally, the samples were dried overnight in a desiccator. The MG samples were mounted on holders using carbon tape and coated with a thin carbon layer to enhance conductivity. Subsequently, the microstructure and elemental composition of the samples were analyzed with a scanning electron microscope (SEM, Apreo S, Thermo Fisher Scientific, USA) equipped with an energy-dispersive X-ray spectroscopy (EDS) detector.

### 2.5 Fourier Transform Infrared Spectroscopy

The chemical structures of the raw materials and the fabricated MG were investigated using Fourier transform infrared spectroscopy equipped with an attenuated total reflectance accessory (ATR-FTIR, Tensor 27, Bruker, USA). For the analysis, freeze-dried MGs, as well as GG and SA, were used in powder form and placed directly onto the ATR crystal. The liquid components, SPAN80 and canola oil, were analyzed by applying a small drop onto the diamond crystal surface. Spectra were acquired for each sample in the wavenumber range of 4000 to 400 cm^–1^. Each spectrum represents an average of 32 scans recorded at a resolution of 1 cm^–1^. The collected data were processed using OPUS software (version 7.2, Bruker).

### 2.6 Thermogravimetric Analysis

The thermal stability and decomposition profiles of the samples were evaluated by thermogravimetric analysis (TGA; TGA 550, TA Instruments, USA). For each measurement, approximately 5 mg of a freeze-dried sample was placed in an open platinum pan and heated from 40 to 600 °C at a constant heating rate of 10 °C/min. The analysis was carried out under a dynamic nitrogen atmosphere with a flow rate of 30 mL/min. The resulting weight loss data were recorded using TRIOS software (Version 4.3.1).

### 2.7 X- ray Diffraction

X-ray diffraction (XRD) analysis was performed to determine the crystalline phases present in the mineralized MG and, for comparison, the nonmineralized MG, as well as a control sample of brushite precipitate. The freeze-dried and powdered samples were placed in a low-background sample holder. XRD patterns were obtained using an X-ray diffractometer (Philips X’Pert system, PANalytical, Malvern, UK) with Cu Kα radiation (λ = 1.540598 Å). Data were collected over a 2θ range of 5° to 70° with a step size of 0.008° and a counting time of 20 s/step.

### 2.8 Determination of Total Calcium Content

To quantify the total calcium reservoir within the MG, samples were analyzed using flame atomic absorption spectrometry (F-AAS). Briefly, 200 mg of freeze-dried MG from each group (NMM, MM50, MM100) were weighed into Teflon vessels. The samples were mineralized using a wet digestion method in a mixture of concentrated nitric acid (65% HNO_3_) and hydrogen peroxide (perhydrol, 30% H_2_O_2_). The process was carried out in a closed microwave-assisted digestion system (Magnum II Mineralizer, ERTEC, Wrocław, Poland) for 210 min. The resulting colorless solutions were quantitatively transferred to quartz evaporators and evaporated on a hot plate at 120 °C to near dryness. The residue was then diluted in 10 mL volumetric flasks using quadruple-distilled water. Prior to the F-AAS analysis, the samples were further diluted 10-fold. The calcium content was quantified using an iCE3500 spectrometer (Thermo Scientific, Gloucester, UK). The absolute mass of calcium was calculated and expressed as milligrams of calcium per gram of dry sample (mg/g).

### 2.9 Calcium Release Profile

The release of ions from the MG was evaluated by immersing weighed freeze-dried samples (NMM, MM50, and MM100) in Milli-Q water at a concentration of 1000 mg/L. The suspensions were incubated at 37 °C under constant agitation (VX-200 Labnet Vortex Mixer, 30 rpm). At predetermined time points (24 and 72 h), the supernatants were collected and filtered. The analysis of calcium (Ca) concentrations was performed using an Inductively Coupled Plasma Optical Emission Spectrometer (ICP-OES, ICPE-9820, Shimadzu, Japan). The instrument was operated in axial view mode at a radio frequency power of 1.20 kW, with the plasma gas flow maintained at 10.00 L/min. The analytical wavelength selected for detection was 317.933 nm for Ca. Data acquisition was carried out with an exposure time of 30 s per replicate. Quantification was performed using calibration curves prepared by serial dilution of high-purity multielement stock standards (1000 mg/L, Merck, Germany).

### 2.10 Degradation Study

To assess the structural stability of the fabricated MG, a degradation test was performed. MGs were immersed in Minimum Essential Medium (MEM, PAN BIOTECH, Germany) supplemented with 10% fetal bovine serum and incubated at 37 °C. To monitor morphological integrity and macroscopic degradation over time, the samples were observed and imaged at predetermined time points (days 1, 3, 7, and 14) using an inverted optical microscope, equipped with phase contrast (ZEISS Axiovert 40 CFL, Germany).

### 2.11 Biological Evaluation

The cell response to the MG was evaluated using MG63 osteoblast-like cells (ECACC, Salisbury, UK). Cells were maintained in MEM, supplemented with 10% fetal bovine serum, 1% penicillin-streptomycin, and 0.1% sodium pyruvate (PAA, Austria). All cultures were incubated at 37 °C in a humidified atmosphere containing 5% CO_2_. All MGs were freeze-dried and immersed in MEM.

Two experimental setups were employed to assess both the cytocompatibility of MGs and the direct cell–material interaction. In the first setup, MG63 cells were seeded in standard tissue culture polystyrene 48-well plates (SARSTEDT, Germany) at a density of 30,000 cells/well in 0.5 mL of medium and allowed to adhere for 24 h. Subsequently, the medium was replaced with a suspension of MG (NMM, MM50, MM100) in MEM to yield their final concentrations of 250 μg/mL and 500 μg/mL. Cells were cultured in these conditions for 1 and 3 days.

To evaluate cell adhesion on the MG surface, a dynamic culture system was established. Experiments were conducted in 24-well nonadhesive culture plates (SARSTEDT, Germany). Sterile round glass coverslips were used as a positive control for cell adhesion. MGs (NMM, MM50, MM100) were suspended in MEM at concentrations of 250 μg/mL and 500 μg/mL and added to the wells. Afterward, cell suspension was poured directly into the wells containing MG or control coverslips at a density of 30,000 cells/well in 1 mL of medium. To ensure homogeneous nutrient distribution and promote cell-MG contact, the plates were placed on a rocker shaker (Rocker 3D digital, IKA, Germany) set to 12 rpm within the incubator. Cell cultures were maintained for 1 and 3 days prior to analysis.

For the AlamarBlue (AB) assay, a 5% AB reagent (Sigma-Aldrich, Germany) was prepared in MEM. The culture medium was removed from the wells, and 0.5 mL for the static experiment and 1 mL for the dynamic experiment of the prepared solution were added to an empty well to establish 0% reduction, as well as to the cell-containing wells, followed by incubation for 2 h at 37 °C in a 5% CO_2_ atmosphere. After incubation, 100 μL of the supernatant was transferred to a black 96-well plate (ThermoFisher Scientific, USA) for fluorescence measurement (λ_ex_ = 544 nm, λ_em_ = 590 nm, FluoStar Omega, BMG Labtech, Germany). To determine a 100% reduction, resazurin was completely reduced using an autoclave. The percentage of resazurin reduction was then calculated using eq 1:

1 $$\%\mathrm{resazurin}\;\mathrm{reduction}=\frac{Rx-R_{b}}{R_{r}-R_{b}}\times 100\%$$

where *R*_*x*_ is the fluorescence of the samples, *R*_*b*_ is the fluorescence of MEM with 10% AB reagent, without cells (0% reduction of resazurin), and *R*_*r*_ is the fluorescence of MEM with 10% AB reagent, autoclaved at 121 °C for 15 min (100% reduction of resazurin).

For live/dead staining, a solution of Calcein AM and propidium iodide, 0.1% v/v each, was prepared in Dulbecco’s phosphate-buffered saline (DPBS, PAN BIOTECH, Germany). The culture medium was removed, and 200 μL of the prepared staining solution was added to each well, followed by incubation in the dark for 20 min. After incubation, fluorescence microscopy images were acquired (ZEISS Axiovert 40 CFL with metal halide illuminator, Germany).

To visualize cell spreading and cytoskeletal organization in the dynamic culture setup, we performed F-actin staining. After 1 and 3 days, cells were washed with PBS, fixed with 4% paraformaldehyde (PFA, Sigma-Aldrich, Germany) for 15 min, and permeabilized with 0.1% Triton X-100 for 5 min. The actin cytoskeleton was stained using phalloidin conjugated with Alexa Fluor 488 Phalloidin (Thermo Fisher Scientific, United States), and nuclei were counterstained with DAPI (Sigma-Aldrich, Germany). Samples were imaged using fluorescence microscopy (ZEISS Axiovert 40 CFL with a metal halide illuminator, Germany) to assess cell morphology and interaction with the MG surface. Additionally, to investigate the cell-material interaction in 3D at high resolution, the samples were imaged by using a confocal microscope (ZEISS LSM 900, Germany). Z-stack images were acquired to visualize the spatial arrangement of the actin cytoskeleton on the MG surface.

### 2.12 Statistical Analysis

Data are presented as the mean ± standard deviation of three independent experiments with three replicates each. Statistical significance between groups was determined using one-way ANOVA followed by Tukey’s post hoc test for multiple comparisons. All statistical analyses were performed using Origin software (version 2022Pro SR1, OriginLab Corporation, Northampton, MA, USA). A probability value less than 0.05 was considered statistically significant. Significance levels are indicated as *p < 0.05, ** *p* < 0.01, and *** *p* < 0.001.

## 3 Results

### 3.1 Morphology and Size Distribution of As-Received Microgels

The fabrication process was successfully optimized to handle the high viscosity of the gellan gum solutions, achieving a final MG yield of 85%. Material losses were minimal and primarily associated with the transfer of the emulsion and the washing steps. The experimental parameters were critical for establishing a stable emulsification window. A processing temperature of 60 °C was selected as it ensured sufficient fluidity for stable extrusion; lower temperatures resulted in premature gelation and nozzle clogging. Regarding the emulsification time, 60 min was found to be necessary to achieve a stable and monodisperse population. Shorter mixing periods yielded polydisperse MG populations with larger average diameters, whereas extending the time beyond 60 min did not result in any further significant reduction in particle size.

The morphology and size of the as-received MG were evaluated using optical microscopy (Figure 3). NMMs (Figure 3A) were characterized by a regular, spherical shape, a smooth surface, and high transparency. Unlike the control samples, MM50 (Figure 3B) and MM100 (Figure 3C) were nontransparent and exhibited a noticeable granular internal structure. Visual differences were observed depending on the concentration of the phosphate precursor; the MM100 MG (Figure 3C) appeared to be less transparent and more densely filled with the inorganic phase than the MM50 MG (Figure 3B). All fabricated particles, regardless of their compositions, maintained a spherical morphology.

**Figure 3:**
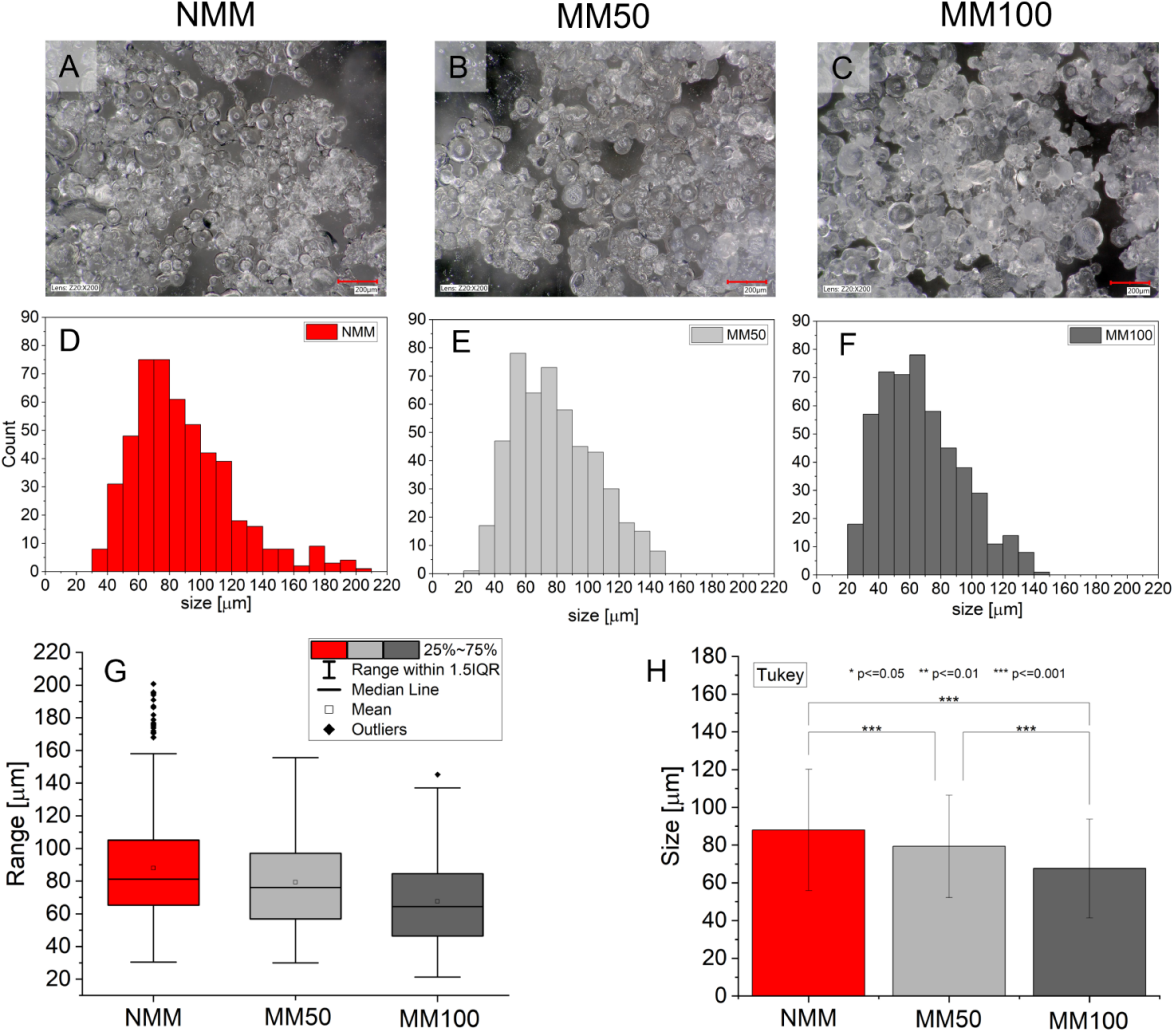
Optical microscopy images of the produced microgels (MGs): nonmineralized (NMM) (A); mineralized with 50 mM Na_2_HPO_4_ (MM50) (B); mineralized with 100 mM Na_2_HPO_4_ (MM100) (C). (A)–(C) Scale bar = 200 μm. Histograms of MG size distribution, *n* = 500 (D–F). Statistical analysis of particle size distribution, box plot (G), and comparison of mean MG sizes (H), *p* ≤ 0.001, according to one-way ANOVA followed by Tukey’s posthoc test.

A statistical analysis of the particle size distribution was performed for all three samples (NMM, MM50, and MM100). The key descriptive statistics for each group are summarized in Table 1.

**Table 1:** Key Descriptive Statistics Characterizing the Particle Size Distribution in the Studied Samples, *n* = 500 for Each Type of Microgels (MGs): Non-Mineralized (NMM), Mineralized with 50 mM Na_2_HPO_4_ (MM50), Mineralized with 100 mM Na_2_HPO_4_ (MM100)^a^

Sample	Mean ± SD [μm]	Median [μm]	Skewness
NMM	88 ±32	81	1.02
MM50	79 ± 27	76	0.51
MM100	68 ± 26	65	0.54

a Statistical significance between groups was confirmed by one-way ANOVA followed by Tukey’s posthoc test (*P* < 0.001).

A clear decreasing trend was observed for both the mean particle size and standard deviation in the series NMM > MM50 > MM100 (Table 1). The shape of the distributions, presented in the histograms (Figure 3D–F), was asymmetric with a distinct right-sided skew, which was confirmed by the positive values of the skewness coefficients (Table 1). The highest asymmetry (1.02) was observed for the NMM sample, which was associated with the presence of numerous outliers, as visualized in the boxplot (Figure 3G).

To formally compare the mean particle sizes between the groups, a one-way analysis of variance (ANOVA) was conducted. A posthoc analysis using Tukey’s HSD test confirmed that all three groups differ from each other in a highly significant manner (*p* < 0.001 for all pairwise comparisons), as graphically represented in Figure 3H.

### 3.2 Microstructure and Chemical Composition of Microgels in Dried Form

The microstructure and surface morphology of the dried MG were examined using SEM (Figure 4A–C), while their elemental composition was determined by EDS (Figure 4D–F). The analysis revealed morphological differences between the nonmineralized and mineralized samples. The dried NMM MGs appeared as collapsed, wrinkled structures with a relatively smooth surface (Figure 4A). In contrast, the mineralized MGs, MM50 (Figure 4B) and MM100 (Figure 4C), largely retained their spherical shape throughout the preparation process. Their surfaces were more textured and appeared to be porous, clearly indicating the presence of a mineral phase. The surface of MM100 appeared denser and more compact compared with the more fibrous texture of MM50.

**Figure 4:**
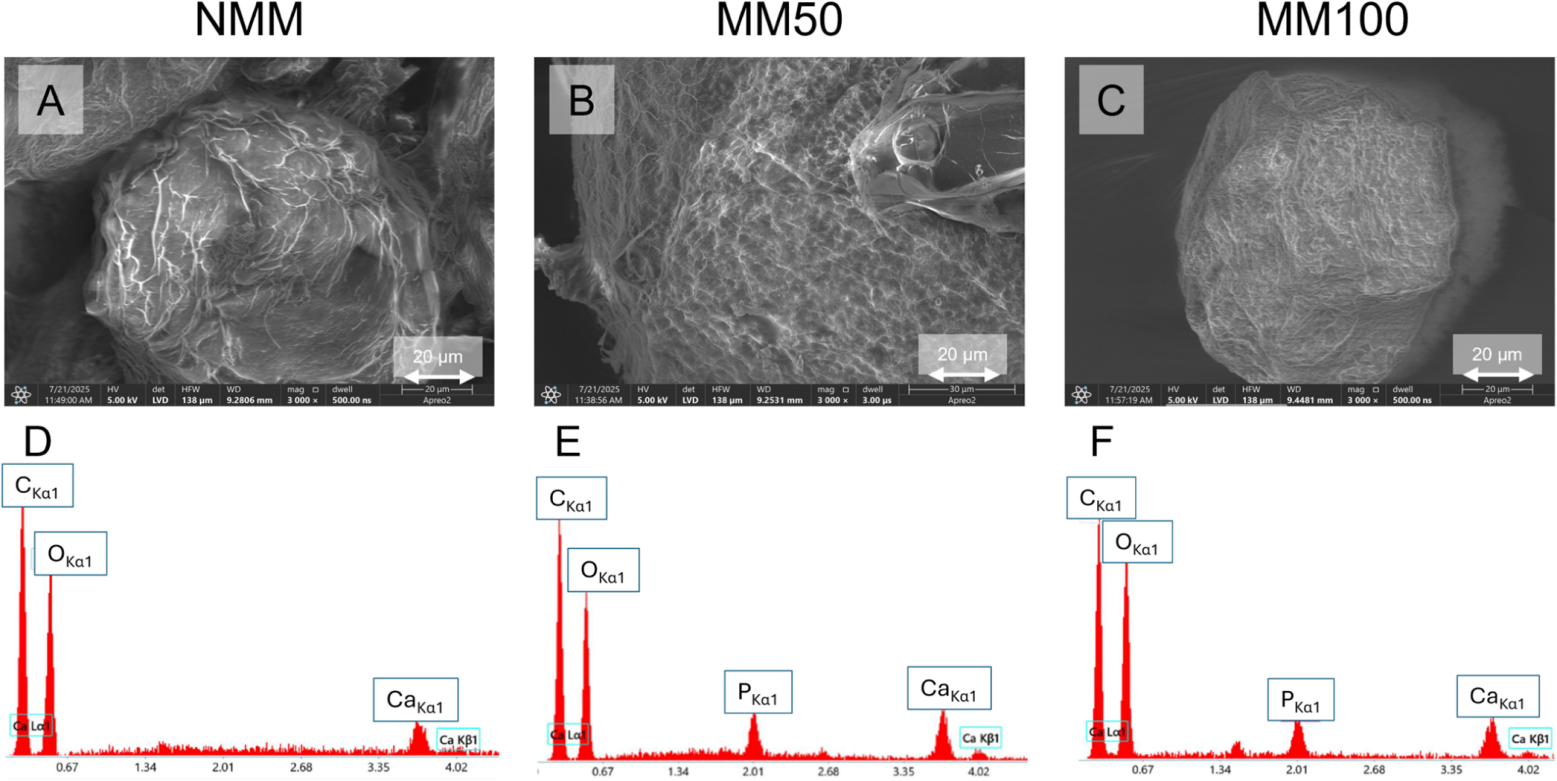
SEM micrographs of dried microgels (MGs): nonmineralized (NMM) (A), mineralized with 50 mM Na_2_HPO_4_ (MM50) (B), and mineralized with 100 mM Na_2_HPO_4_ (MM100) (C). EDS analysis of the elemental composition: NMM (D), MM50 (E), and MM100 (F) samples.

The EDS elemental analysis (Figure 4D–F) confirmed these morphological findings. The spectrum of the NMM sample showed dominant peaks for carbon (C) and oxygen (O), originating from the polysaccharide matrix; the presence of C is also due to carbon layer sputtering to make the sample conductive for SEM analysis. Moreover, in NMM, only a trace amount of calcium (Ca) from the CaCl_2_ used as a cross-linking agent for GG/SA hydrogels is present; no signal from phosphorus (P) was detected. On the contrary, the spectra for MM50 and MM100 samples clearly displayed intense peaks for both calcium and phosphorus, providing unambiguous evidence for the incorporation of a calcium phosphate phase. The atomic ratio Ca/P for MM50 was 2.00, while for MM100, it was 1.78.

### 3.3 Fourier Transform Infrared Spectroscopy

The chemical structures of the raw materials and the final MG formulations were analyzed by ATR-FTIR, with the resulting spectra being presented in Figure 5.

**Figure 5:**
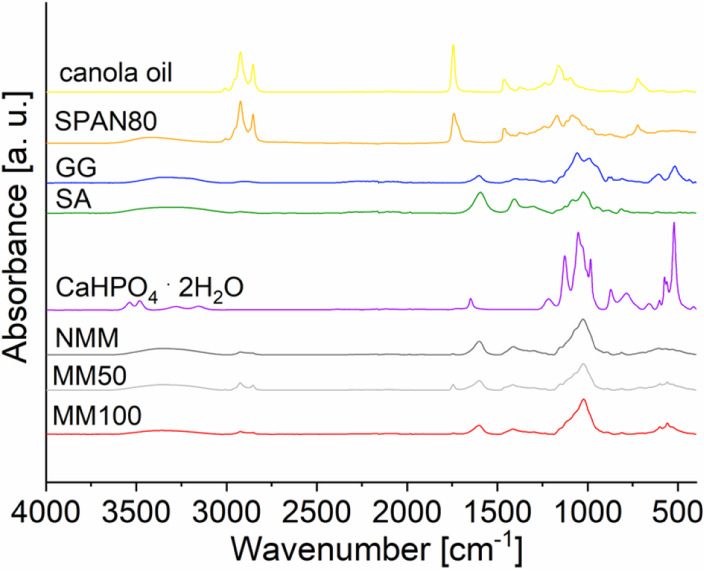
ATR-FTIR spectra of microgels (MGs): nonmineralized (NMM), mineralized with 50 mM Na_2_HPO_4_ (MM50), mineralized with 100 mM Na_2_HPO_4_ (MM100), reference gellan gum (GG), sodium alginate (SA), brushite (CaHPO_4_·2H_2_O), and the components of the oil phase (canola oil, SPAN80).

The FTIR spectra of the raw polysaccharides (GG and SA) were characterized by a broad band in the 3600–3000 cm^–1^ region, attributed to O–H stretching vibrations.^47^ Two intense bands, characteristic of carboxylate groups, that were observed at 1600 cm^–1^ and 1415 cm^–1^ were assigned to asymmetric and symmetric stretching vibrations of (COO−) groups, respectively.^48^ The spectrum of pure brushite (CaHPO_4_·2H_2_O) revealed bands for both phosphate groups and water of crystallization. An intense, sharp band at 1120 cm^–1^ was attributed to the asymmetric stretching vibrations of the P–O bond, and a sharp peak at 555 cm^–1^ corresponded to the O–P–O bending vibrations in the phosphate group. The presence of hydration water was confirmed by a broad band in the 3550–3200 cm^–1^ region from O–H stretching and a band at 1650 cm^–1^ originating from H–O–H bending.^49^

The spectrum of the NMM displayed all the characteristic bands of the polysaccharide matrix. For all MG samples (NMM, MM50, and MM100), the absorption band at 1745 cm^–1^, attributed to C=O stretching vibrations in ester groups characteristic of the oil and surfactant, was observed at a negligible intensity. This confirms the effective removal of residual oil and surfactant phases by the multistep washing procedure.

The spectra of the mineralized MG (MM50 and MM100) were a clear superposition of the polymer and mineral phase spectra. Evidence of mineralization was provided by the appearance of a new, sharp peak at 555 cm^–1^, which was absent in the NMM spectrum and corresponds to the characteristic O–P–O bending vibration of the brushite standard.^50^ While other changes were noted in the 1200–800 cm^–1^ region, they were partially obscured by the strong band from C–O stretching vibrations in the polysaccharides. The intensity of the peak at 555 cm^–1^ was notably higher in the MM100 sample compared to the MM50 sample.

### 3.4 Thermogravimetric Analysis

The thermal stability of all the MG and raw brushite was investigated using TGA, with the results presented as weight loss curves (TGA) and derivative thermogravimetric curves (DTG) in Figure 6.

**Figure 6:**
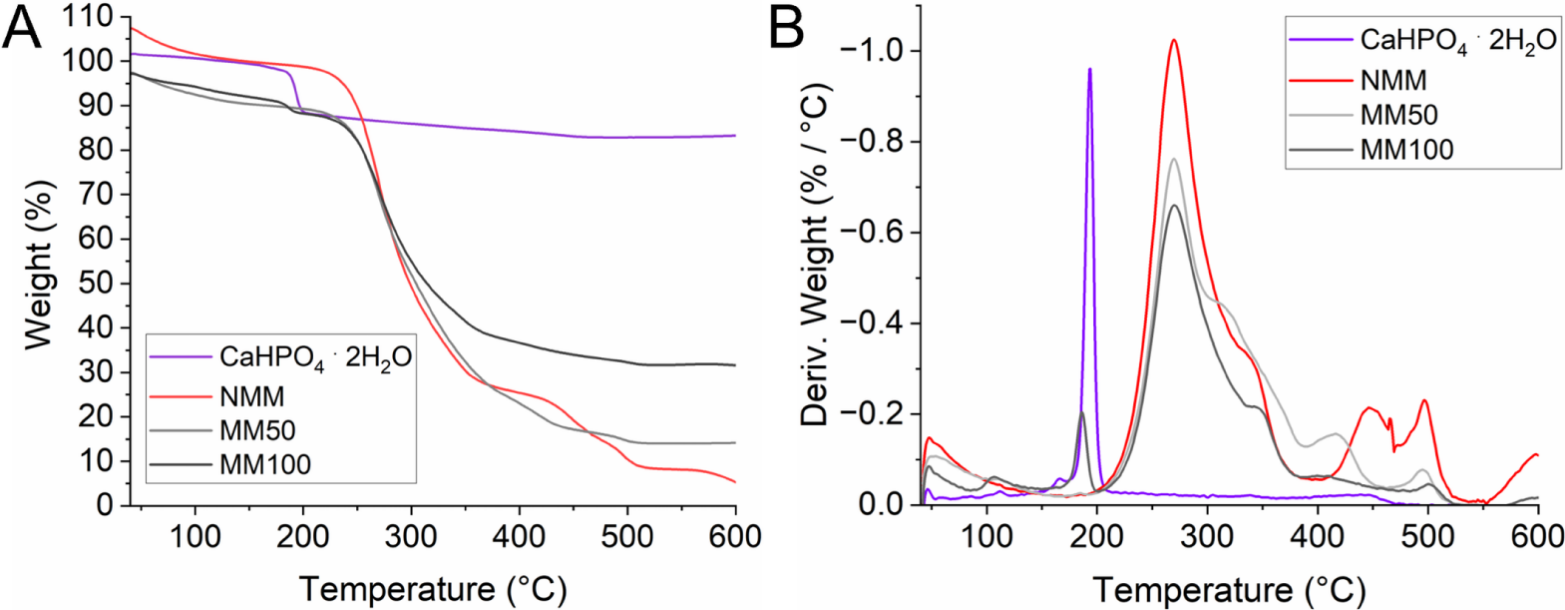
Thermogravimetric TG (A) and derivative thermogravimetric DTG (B) curves for the pure brushite standard (CaHPO_4_·2H_2_O) and microgels (MG): nonmineralized (NMM), mineralized with 50 mM Na_2_HPO_4_ (MM50), and mineralized with 100 mM Na_2_HPO_4_ (MM100).

Pure brushite (CaHPO_4_·2H_2_O) exhibited a distinct weight loss of approximately 16% between 150 and 220 °C, corresponding to the loss of its two crystalline water molecules, followed by high thermal stability up to 600 °C. The NMM showed a major thermal decomposition event starting around 220 °C, with the maximum rate of weight loss occurring at approximately 280 °C, as indicated by the sharp peak on the DTG curve. The final residual mass for NMM at 600 °C was low, at around 9%.

The mineralized MGs (MM50 and MM100) showed composite thermal behavior. Their DTG curves featured a peak around 200 °C, corresponding to the dehydration of the brushite phase, as well as the peak of the main polymer decomposition between 270 and 280 °C. The mineralized samples exhibited a significantly higher residual mass at 600 °C compared to the NMM control. The final residue was approximately 18% for MM50 and increased substantially to approximately 35% for MM100.

### 3.5 X-ray Diffractometry

The crystalline phase of the incorporated minerals was identified by XRD (Figure 7). For this analysis, the MM100 sample was selected as a representative of the mineralized group due to its significantly higher mineral content, as confirmed by TGA and EDS, ensuring a better signal-to-noise ratio.

**Figure 7:**
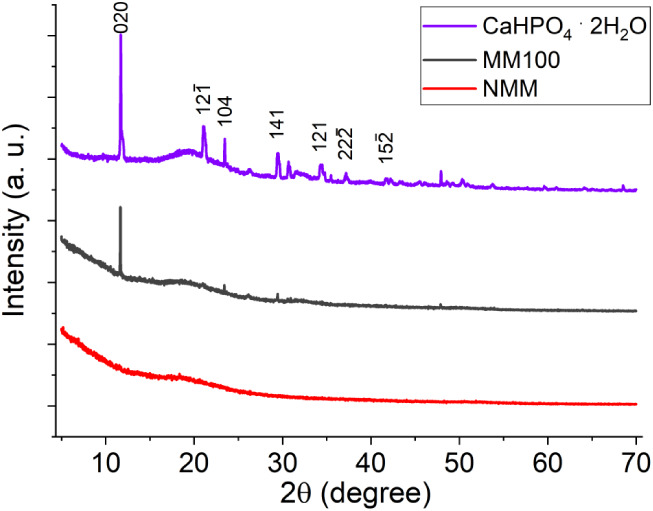
X-ray diffraction patterns of the pure brushite (CaHPO_4_·2H_2_O), nonmineralized microgels (NMM), and mineralized with 100 mM Na_2_HPO_4_ microgels (MM100).

The diffractogram of the NMM showed a single, broad amorphous halo centered around 2θ ≈ 22°, which confirms the amorphous nature of the polysaccharide matrix. On the contrary, the pure CaHPO_4_·2H_2_O standard exhibited a pattern with numerous sharp and intense diffraction peaks at 2θ ≈ 11.6° (020), 20.9° (121̅), 23.4°(104), 29.2° (141), confirming its highly crystalline brushite structure.^51^

The XRD pattern of the MM100 sample displayed features of both components: a broad amorphous hump from the polymer matrix, overlapped with several distinct, although less intense, diffraction peaks. Importantly, the positions of these peaks in the MM100 sample, particularly the most prominent peak at 2θ ≈ 11.6° and the smaller peak at 2θ ≈ 23.4°, perfectly matched the characteristic peaks of the pure brushite standard.^52^ This result confirms that the mineral phase precipitated *in situ* within the MG is crystalline brushite.

### 3.6 Total Calcium Content

The total calcium content of the MG was quantified via F-AAS to determine the initial mineral reservoir, expressed as mg per g of dry sample (Figure 8). The baseline calcium content in the NMM group was 38.1 ± 0.7 mg/g. In the mineralized groups, the total calcium loading was significantly higher, determined to be 54.1 ± 1.2 mg/g for MM50 and 76.1 ± 1.9 mg/g for MM100.

**Figure 8:**
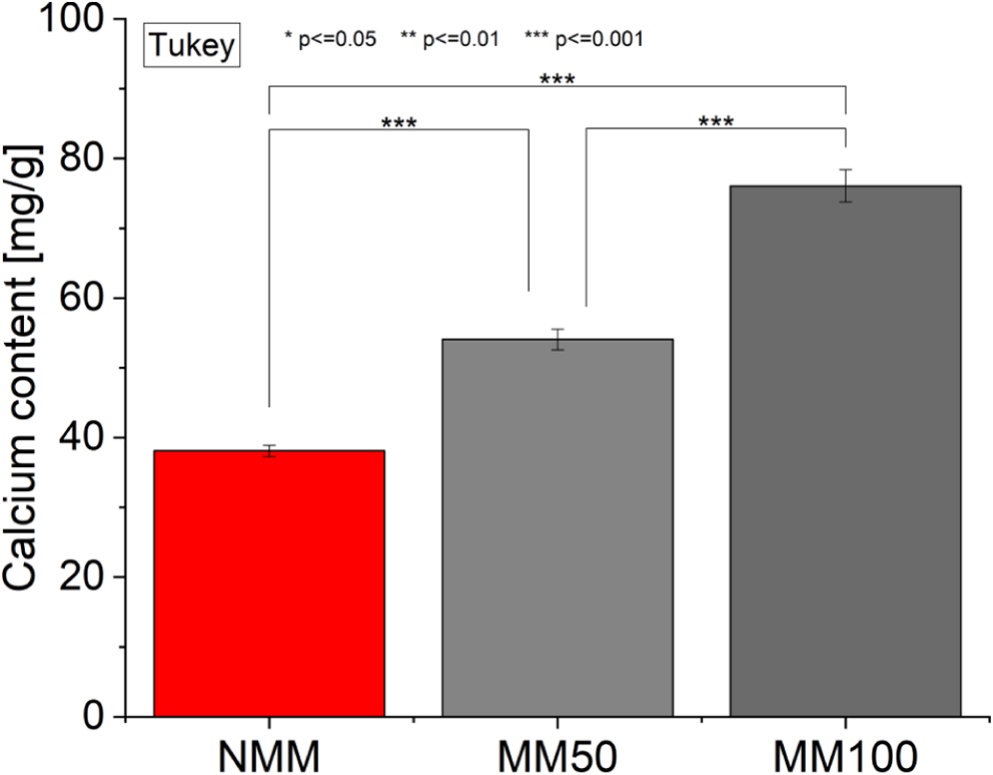
Total calcium content in the fabricated microgels (MGs): nonmineralized (NMM), mineralized with 50 mM Na_2_HPO_4_ (MM50), and mineralized with 100 mM Na_2_HPO_4_ (MM100), expressed as mg/g of dry sample. Statistical significance between groups was confirmed by one-way ANOVA followed by Tukey’s posthoc test (*** *p* ≤ 0.001).

### 3.7 Calcium Release Profile

The cumulative release of Ca^2+^ from the MG is presented in Figure 8. As expected, the NMM showed negligible calcium release, originating only from calcium playing the role of the cross-linking agent. In contrast, the mineralized samples demonstrated a sustained release of Ca^2+^. The MM100 MG released significantly higher amounts of calcium compared to MM50, reaching a concentration of 17.9 ± 0.1 mg/L after 24 h and increasing to 24.4 ± 0.1 mg/L after 72 h. This confirms that the brushite phase embedded within the hydrogel matrix remains accessible and effectively acts as a source of free Ca^2+^ in an aqueous environment, Figure 9.

**Figure 9:**
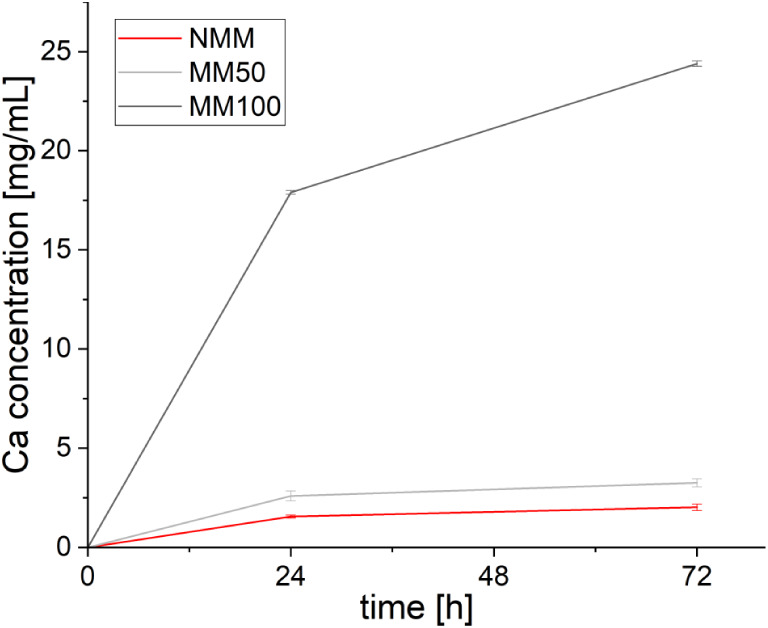
Calcium release profile of fabricated microgels (MGs): nonmineralized (NMM), mineralized with 50 mM Na_2_HPO_4_ (MM50), and mineralized with 100 mM Na_2_HPO_4_ (MM100), incubated in Milli-Q water at a concentration of 1 mg/L for 24 and 72 h.

### 3.8 Degradation and Structural Stability

A degradation study was conducted over a 14-day period in MEM at 37 °C. Optical microscopy evaluation on days 1, 3, 7, and 14 (Figure 10) revealed that the MGs maintained their spherical morphology and physical cohesion throughout the 14-day incubation. The samples resisted macroscopic dissolution and did not exhibit excessive swelling or fragmentation during the observation period.

**Figure 10:**
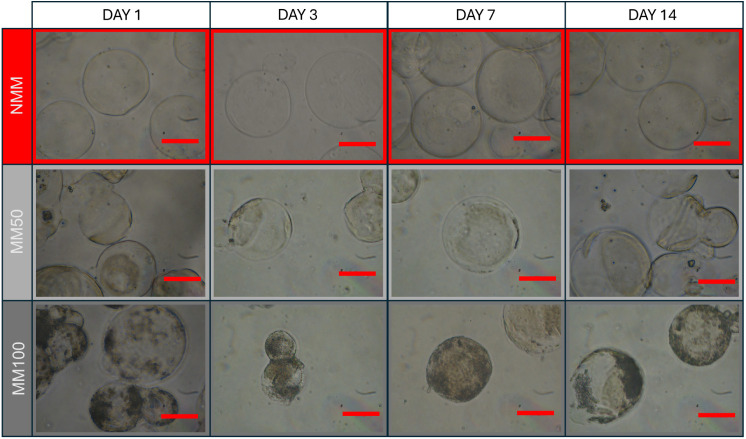
Representative optical microscopy images demonstrating the structural stability of the fabricated microgels (MGs): nonmineralized (NMM), mineralized with 50 mM Na_2_HPO_4_ (MM50), and mineralized with 100 mM Na_2_HPO_4_ (MM100), during the 14-day degradation study. Scale bar = 50 μm.

### 3.9 Biological Evaluation

The metabolic activity of MG63 cells was evaluated in two experimental setups: direct contact in a static culture and direct contact in a dynamic culture. In the first setup (Figure 11A), cells were first cultured in the tissue culture polystyrene multiwell plates for 1 day, and afterward, MGs were added for another 1 and 3 days. After day 1, no statistically significant differences were observed between the control group and any MG-treated groups. Although a significant increase in metabolic activity was observed for all groups on day 3 compared to day 1, the differences between the nonmineralized (NMM) and mineralized samples (MM50, MM100) remained statistically insignificant. Cell viability and morphology assessed after live/dead staining (Figure 11B) confirmed high cytocompatibility among all groups. Most of the cells remained viable (green-stained), with negligible numbers of dead cells (nuclei red-stained).

**Figure 11:**
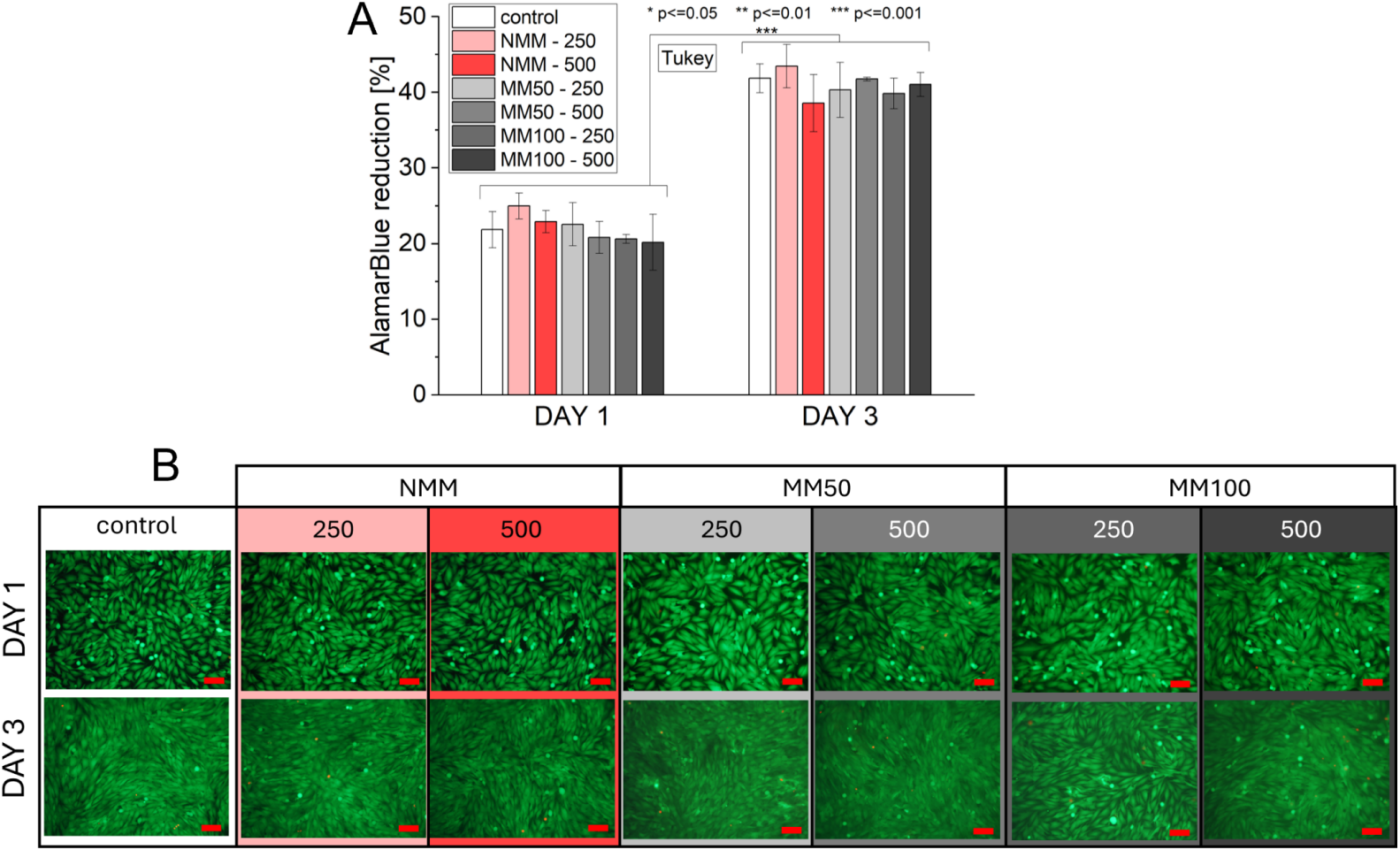
Metabolic activity (A) and live/dead staining (B) of MG63 osteoblast-like cells cultured in direct contact with fabricated microgels (MGs): nonmineralized (NMM), mineralized with 50 mM Na_2_HPO_4_ (MM50), and mineralized with 100 mM Na_2_HPO_4_ (MM100), at concentrations of 250 and 500 μg/mL for 1 and 3 days. *p* ≤ 0.001 according to one-way ANOVA followed by Tukey’s post hoc test. Scale bar = 100 μm applies to all respective images.

In contrast, the dynamic culture setup (Figure 12A), designed to enhance fluid flow, revealed distinct differences dependent on the material composition. While the NMM group showed metabolic activity comparable to the control, both mineralized groups (MM50 and MM100) exhibited significantly higher resazurin reduction values on day 3 compared to the nonmineralized samples. Notably, this elevated metabolic activity was observed in both mineralized groups, regardless of the mineral content.

**Figure 12:**
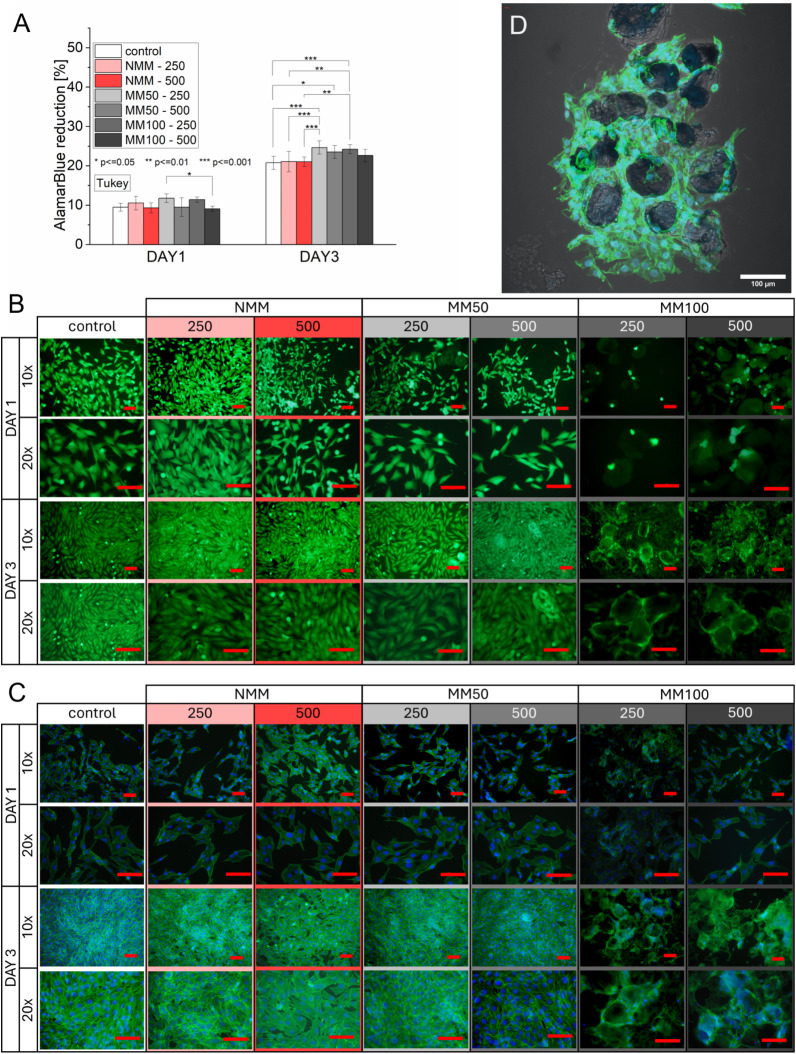
Metabolic activity (A), live/dead staining (B), and phalloidin/DAPI (C) of MG63 osteoblast-like cells cultured in dynamic conditions with fabricated microgels (MGs): nonmineralized (NMM), mineralized with 50 mM Na_2_HPO_4_ (MM50), and mineralized with 100 mM Na_2_HPO_4_ (MM100), at concentrations of 250 and 500 μg/mL for 1 and 3 days. Phalloidin/DAPI-stained cells on confocal microscopy at concentrations of 250 μg/mL after 3 days (D). *p* ≤ 0.05, *p* ≤ 0.01, and *p* ≤ 0.001 according to one-way ANOVA followed by Tukey’s post hoc test. Scale bar = 100 μm applies to all respective images.

Fluorescence microscopy analysis under dynamic conditions (Figure 12B–C) provided detailed insight into cell morphology and distribution. In the control samples cultured on nonadhesive plates, a lower cell density was observed on day 1; however, by day 3, the cells had proliferated to form a confluent monolayer, driven by cell–cell interactions. In the experimental groups containing MG, distinct interaction patterns emerged. In the presence of NMM and MM50, cells were found to be primarily settled at the well bottom, forming aggregates in the spaces between the MG. In these groups, the MG themselves remained largely uncolonized. Conversely, a striking difference was observed in the MM100 group. Cells were found to adhere directly to the MG surfaces, clearly outlining the spherical shape of the particles. By day 3, live and dead staining and phalloidin/DAPI staining revealed dense cell coverage on the MM100 MG. High-resolution confocal microscopy (Figure 12D) further confirmed this interaction, showing cells with well-developed actin cytoskeletons tightly enveloping the spherical MM100 MG, in sharp contrast to the planar cell sheets observed in the NMM and MM50 groups.

## 4 Discussion

### 4.1 A Novel Fabrication Approach for Robust, Size-Tunable Spherical Microgels

The primary goal of this research was to overcome a key technological barrier: the fabrication of uniform MGs with potential injectability from materials that are inherently difficult to process, such as high-concentration gellan gum. Conventional methods often fail due to the high viscosity of such solutions at room temperature, leading to nozzle clogging and heterogeneous particle formation.^42^ Our success in producing spherical MGs with a mean diameter below 100 μm directly demonstrates the efficacy of our custom-designed, temperature-controlled emulsification system. By maintaining the polymer solution at 60 °C throughout the extrusion process and homogenization, we effectively prevented premature gelation and ensured consistent droplet formation, thus solving a critical manufacturing problem. However, even with this advanced thermal management, the platform has operational boundaries. We experimentally determined that the concentration of 2.5% (w/v) gellan gum acts as the upper limit for our pumping system. At higher concentrations, the viscosity increases drastically even at 60 °C, which prevents stable flow through the needle and causes irregular extrusion and fragmentation rather than spherical droplet formation. Conversely, lower polymer concentrations (<1%) failed to produce mechanically robust particles after gelation, highlighting the necessity of processing high-concentration solutions for structural stability.

The initial results of optical microscopy provided the first visual evidence of success. A distinct transition was observed from the transparent, purely polysaccharide MG (NMM) to the opaque granular composites (MM50 and MM100), indicating successful *in situ* mineral precipitation. Subsequent SEM analysis offered deeper insight into MG structural integrity. Despite chemical fixation intended to preserve their morphology, the NMM appeared collapsed and wrinkled. In contrast, the mineralized samples remained robustly spherical. This demonstrates that the incorporated mineral phase acts as an effective internal scaffold, providing a shape stability far superior to that of the pure polymer network.

This enhanced structural integrity was accompanied by a statistically significant and systematic decrease in particle size with increasing mineralization. This phenomenon can be attributed to two contributing effects. First, the high ionic strength of the Na_2_HPO_4_ precursor solution screens electrostatic repulsions between the anionic polysaccharide chains, promoting a more compact polymer conformation and thus the formation of smaller initial droplets during emulsification.^53,54^ Second, during the subsequent gelation step, the rapid *in situ* precipitation of brushite probably generates an osmotic gradient that drives the expulsion of water from the forming network, resulting in a network collapse and further shrinkage of the final particles.^53,55–57^ This combined effect not only explains the smaller and denser nature of the mineralized MG but also represents a key formulation advantage as the ability to produce smaller and more uniform particles is highly beneficial for their application as injectable carriers.

### 4.2 Physicochemical Confirmation of a Tunable Nanocrystalline Brushite-Polymer Composite

A comprehensive suite of complementary analyses provided a complete and consistent physicochemical picture of the fabricated materials, confirming their identity and tunability. The presence of a mineral phase, first suggested by microscopy, was unequivocally confirmed by EDS, which identified both calcium and phosphorus exclusively in the mineralized samples. The precise identity of this phase was then elucidated by FTIR and XRD. FTIR served as a powerful molecular fingerprinting tool, with the appearance of a new, sharp peak at 555 cm^–1^ in a region free of bands characteristic of the polymer, providing unambiguous evidence for calcium phosphate. This was definitively confirmed by XRD analysis, which identified the precipitated phase as crystalline brushite with a diffraction peak at 2θ ≈ 11.6°. Based on the Scherrer equation, these results suggest that the broader and less intense diffraction peaks observed in the MM100 sample compared to the pure brushite standard are characteristic of a smaller crystallite size,^58,59^ a desirable feature that may enhance bioactivity and resorption rates, mimicking biological apatite.^60^

The ability to control the mineral content, which is a key goal of this study, was quantitatively demonstrated by TGA. The clear, concentration-dependent trend in residual mass (18% for MM50 vs 35% for MM100) provides robust evidence that the mineral loading can be precisely regulated by adjusting the precursor concentration. Finally, FTIR analysis also served as a vital quality control check, confirming that the washing procedure was highly efficient at removing residual oil and surfactant and ensuring the purity of the final product.

A key advantage of incorporating brushite into the MG structure is its significantly higher solubility compared to more thermodynamically stable calcium phosphates, such as hydroxyapatite.^31^ Previous studies have shown that hydroxyapatite-based composites often act as static fillers due to their low solubility. In contrast, our results align with findings by Bjørnøy et al.,^33^ demonstrating that the brushite phase undergoes faster resorption, thereby creating a bioactive environment rich in Ca^2+^ and phosphate ions. This confirms that selecting brushite over more stable calcium phosphates was crucial for achieving the desired ion-release profile presented in Figure 8. Furthermore, quantifying the total calcium reservoir via F-AAS provided crucial context for these release kinetics. When comparing the substantial internal reservoir of MM100 (76.1 ± 1.9 mg/g) to its cumulative release over 72 h (approximately 24.4 mg/L from a 1 mg/mL suspension), it becomes evident that about 32% of the incorporated calcium is released within the first 3 days. This confirms that the MGs do not exhibit a rapid burst release but rather provide a sustained, controlled release behavior governed by the gradual dissolution of the brushite phase. This sustained release of ionic signals is hypothesized to be the primary driver of material bioactivity, making it a prime candidate to induce osteogenic differentiation of progenitor cells and promote polarization of macrophages toward a proregenerative phenotype M2, as outlined by others.^34–40^

Furthermore, by modulating process conditions, such as pH, temperature, or precursor ratios, the mineralization process could likely be geared toward other bioactive crystalline phases, such as octacalcium phosphate or hydroxyapatite, depending on the desired resorption profile and therapeutic application.

### 4.3 Cytocompatibility and Bioactivity

The primary requirement for any bone-substitute biomaterial is cytocompatibility. The *in vitro* results obtained from both static and dynamic culture models unequivocally establish the safety of the newly developed MG. Qualitative data from live/dead staining, demonstrating high cell viability across all experimental groups, were in excellent agreement with the quantitative AlamarBlue assay, indicating that the metabolic activity and proliferation of osteoblast-like MG63 cells were not compromised by contact with the materials. It is particularly important that both the pure polymer matrix (NMM) and the brushite-mineralized composites (MM50 and MM100) were found to be noncytotoxic. This confirms that the material components, as well as their potential ionic dissolution products, do not elicit an adverse cellular response, successfully passing the initial biological safety screening.

However, the introduction of a dynamic culture environment revealed a crucial decoupling between the chemical bioactivity and structural competence. While the NMM remained bioinert, both mineralized groups (MM50 and MM100) exhibited a statistically significant increase in metabolic activity. This effect is directly attributed to the sustained release of calcium and phosphate ions from the dissolving brushite phase, as confirmed by our ICP-OES analysis (Figure 8). Extracellular Ca^2+^ and inorganic phosphate are potent signaling molecules known to stimulate osteoblast metabolism and proliferation via intracellular pathways,^37,38^ thereby enhancing cellular activity even in the absence of direct cell adhesion.

Critically, our microscopic observations demonstrated that this chemical stimulation does not automatically translate to physical cell adhesion. In the NMM and MM50 groups, cells failed to colonize MG. Instead, driven by the lack of anchorage points on the bioinert gellan gum/alginate surface, the cells favored cell–cell interactions over cell–matrix interactions. This resulted in the formation of cohesive cell sheets or aggregates that settled at the bottom of the wells rather than on the hydrogel surface. Due to the nonfluorescent nature of the hydrogel matrix, the uncolonized MG in the NMM and MM50 groups was not directly visible. In contrast, in the MM100 group, the spherical shape of the MG was clearly visualized by the outline of the adherent cell layer.

The most important aspect of this study is that MM100 MG, characterized by a higher mineral load, supported robust cell adhesion and spreading. The observation of cells around the MM100 MG provides evidence that a sufficient density of the mineral phase is required to provide the necessary surface roughness and stiffness for physical anchorage. This 3D cell-material integration was further substantiated by confocal imaging, which visualized the actin cytoskeleton adapting to the geometry of the mineralized MG. Consequently, while lower mineralization levels may be sufficient to stimulate cell metabolism, a high mineral loading is essential to transform the hydrogel from a passive delivery material into an osteoconductive scaffold capable of supporting 3D cellular assembly.

The current *in vitro* evaluation successfully demonstrates high cytocompatibility and the critical onset of 3D cell–matrix integration on the heavily mineralized MM100 MG within a 3-day dynamic culture time frame. While these initial results are highly promising and validate the bioactive design of MG, future studies will require extended culture periods. Such long-term evaluations will be essential for fully assessing advanced cellular responses, including sustained osteogenic differentiation and *de novo* extracellular matrix deposition.

### 4.4 Significance for Minimally Invasive Therapies

The specific geometry of the fabricated MG offers distinct biological advantages essential for bone tissue engineering. Their micron-scale dimensions result in a high surface-to-volume ratio, which facilitates rapid nutrient and waste transport, thereby sustaining the long-term viability of colonizing cells and improving cell–matrix interactions.^61,62^ Furthermore, the microscale format enhances responsiveness to environmental changes and supports local retention at the injection site, preventing rapid clearance by the host immune system.^63^ While individual MGs are inherently mechanically weaker than bulk macrohydrogels—making them less effective at preserving tissue shape on their own—their collective assembly into a cohesive microgranular hydrogel mitigates this drawback.^64^ This “jammed” granular architecture harnesses the benefits of improved structural stability while retaining the favorable diffusion rates characteristic of microscale systems.

The claim of structural stability is directly supported by the 14-day degradation study. The ability of the mineralized MG to resist rapid dissolution and maintain their spherical integrity in a cell culture medium confirms that they can act as prolonged physical support. Rather than prematurely degrading upon ion exchange, this composite matrix effectively functions as a durable template, which is essential for maintaining volume and structural integrity within a bone defect. Importantly, while the sustained release of calcium ions confirms the dynamic chemical bioactivity of the MG, it does not compromise their physical integrity. As demonstrated by the 14-day degradation study, the mineralized MG successfully maintains its spherical morphology and structural cohesion over time. This indicates that, despite the ongoing partial dissolution of the brushite phase, the remaining mineral content, interlocked within the robust GG/SA matrix, provides sufficient internal scaffolding to prevent the collapse of the particles. Consequently, the MG successfully balances dynamic ion exchange with the long-term structural stability required to act as a durable template for initial cellular colonization and tissue ingrowth.

To fully appreciate the potential of the developed material, it is crucial to contextualize the brushite-mineralized GG/SA system within the broader landscape of hydrogel microparticles. While various polymers have been explored for bone tissue engineering, each presents distinct trade-offs. For instance, gelatin methacrylate microspheres are widely investigated due to their inherent cell-adhesion motifs, yet they typically require chemical modification and photo-cross-linking, which adds complexity compared to the straightforward ionic and thermal gelation employed in our system.^62^ Similarly, elastin-like protein (ELR) MGs have demonstrated osteogenic potential but often achieve lower mineral contents, approximately 20 wt %.^65^ In comparison, our GG/SA MGs successfully incorporate up to ∼35 wt % of the mineral phase. This superior mineral loading is hypothesized to provide enhanced structural stability and a larger reservoir of bioactive calcium and phosphate ions, addressing the mechanical limitations often associated with purely protein-based hydrogels.

## 5 Conclusion

This study successfully establishes an accessible and highly customizable fabrication platform based on precise temperature-controlled emulsification. This approach effectively overcomes the technological challenges associated with processing high-concentration, highly viscous polysaccharide solutions into robust, injectable-sized MGs. Importantly, this platform technology can be successfully applied not only to GG but also broadly to other biopolymers whose rheological properties and gelation mechanisms strongly depend on temperature. Our comprehensive characterization confirmed the formation of a spherical GG/SA MG containing a defined amount of crystalline brushite. Crucially, biological evaluation under dynamic conditions highlighted the functional necessity of the mineral phase: while the pure polysaccharide matrix remained bioinert, the mineralized MG supported robust cell adhesion and cytoskeletal spreading. These findings, combined with the confirmed sustained release of Ca^2+^, establish this material as a highly promising candidate for bone regeneration. The verified bioactivity paves the way for *advanced in vitro* studies to assess the potential to induce osteogenic differentiation and modulate macrophage polarization, ultimately leading to its evaluation in preclinical *in vivo* models of bone healing.

## Abbreviations

GG: gellan gum
SA: sodium alginate
MG: microgels
NMM: nonmineralized microgels
MM50: mineralized microgels with Na_2_HPO_4_ at 50 mM concentration
MM100: mineralized microgels with Na_2_HPO_4_ at 100 mM concentration
